# Identifying Demographic, Clinical, Muscular and Histological Factors Associated with Ultrasound Cervical Multifidus Measurement Errors in a Chronic Neck Pain Population

**DOI:** 10.3390/s22218344

**Published:** 2022-10-31

**Authors:** Juan Antonio Valera-Calero, Marcos José Navarro-Santana, Gustavo Plaza-Manzano, César Fernández-de-las-Peñas, Ricardo Ortega-Santiago

**Affiliations:** 1 VALTRADOFI Research Group, Department of Physiotherapy, Faculty of Health, Universidad Camilo José Cela, Villanueva de la Cañada, 28692 Madrid, Spain; 2 Faculty of Health, Universidad Católica de Ávila, C/Canteros, s/n, 05005 Ávila, Spain; 3 Department of Radiology, Rehabilitation and Physiotherapy, Universidad Complutense de Madrid, 28040 Madrid, Spain; 4 Instituto de Investigación Sanitaria San Carlos (IdISSC), 28040 Madrid, Spain; 5 Department of Physical Therapy, Occupational Therapy, Rehabilitation and Physical Medicine, Universidad Rey Juan Carlos, 28922 Alcorcón, Spain; 6 Cátedra Institucional en Docencia, Clínica e Investigación en Fisioterapia: Terapia Manual, Punción Seca y Ejercicio Terapéutico, Universidad Rey Juan Carlos, 28922 Alcorcón, Spain

**Keywords:** ultrasound imaging, measurement error, reliability, cervical multifidus, neck

## Abstract

Ultrasound imaging (US) is a widely used imaging tool in physiotherapy for assessing muscle morphology and quality, among other purposes, such as ensuring the patients’ safety during invasive procedures or providing visual feedback during motor control exercises. Identifying factors associated with measurement errors is essential to target avoid bias in high-risk of bias populations. Therefore, this study aimed to assess whether demographic, clinical, muscular and histological factors are associated with ultrasound measurement errors in patients with idiopathic chronic neck pain. B-mode images were acquired and analyzed in 126 patients with chronic neck pain by two experienced examiners. Cross-sectional area, muscle perimeter, mean echo intensity and percentage of fatty infiltration were analyzed. The interexaminer agreement was assessed by calculating the absolute error, intraclass correlation coefficient (ICC), standard error of measurement (SEM) and minimal detectable changes (MDC). A Pearson’s correlation matrix including all variables was calculated to conduct a multivariate linear stepwise regression model for estimating the explained variance for each measurement error. Results demonstrated excellent reliability (ICC = 0.965) for assessing the cross-sectional area, and good reliability for assessing the muscle perimeter, mean echo intensity and intramuscular infiltrates estimation (ICC = 0.898, 0.882 and 0.758, respectively). Although clinical variables were not associated with measurement errors (*p* > 0.05), multiple correlations were found between demographic and cervical multifidus characteristics with measurement errors.

## 1. Introduction

The use of ultrasound imaging (US) is currently increasing among physiotherapists as is a portable, safe, accessible and low-cost (in comparison with other imaging methods) tool for a wide variety of purposes [1]. Currently, physiotherapists access US equipment for guiding invasive procedures in order to ensure the patients’ safety [2], as a visual feedback tool for guiding motor control exercises [3] and as an instrument for evaluating and monitoring musculoskeletal structures [4].

In addition, since there are multiple imaging modes based on US technologies, multiple parameters can be easily and objectively assessed. For instance, the Doppler mode is useful for assessing vascular flows (facilitating the identification of vascular structures or assisting with the interventions’ effects on these vessels) [5], the M-mode assists with analyzing thickness changes in real-time (especially useful during motor control exercises if patients need feedback to ensure a correct muscle activation) [6], shear-wave elastography allows an objective assessment of a tissue’s stiffness [7] and panoramic US allows the measurement of large structures not measurable with conventional B-mode images [8].

In addition, offline DICOM software also expands the utility of US images for analyzing histological characteristics, besides muscle morphology or activation assessments [9]. In chronic neck pain populations, there is an increasing interest in identifying histological adaptations associated with pain intensity, pain duration and disability in superficial and deep cervical muscles [10,11,12,13,14].

However, most of the imaging studies assessing cervical muscles use magnetic resonance imaging (MRI) instead of ultrasound imaging since the US reliability in patients with chronic neck pain is still controversial [15,16]. Since reliability estimates reported in the literature for assessing deep neck extensors with US are different in asymptomatic and clinical populations, there is a need for identifying which factors contribute to US measurement error since the hypotheses provided by the authors have not been tested yet. 

Therefore, the aim of this study was to conduct a reliability study recruiting patients with mechanical chronic neck pain and analyze the correlation between demographic, clinical, muscular and histological factors, with the error of measurements, conducting a correlation analysis with a stepwise multivariate linear regression model for explaining the individual contribution of each variable in the error variance. 

## 2. Materials and Methods

### 2.1. Study Design

This study consisted of a cross-sectional observational study design to calculate those demographic (i.e., age, height, weight and body mass index), muscular (i.e., muscle cross-sectional area and perimeter assessed with ultrasound), clinical (i.e., neck disability), and histological (i.e., muscle quality assessed as mean echo intensity and intramuscular fatty infiltration percentage following a procedure previously described in the literature [17]) factors associated with US measurement errors while assessing the cervical multifidus CSA, perimeter, echo intensity and fatty infiltration estimation. Since this study can be considered a diagnostic accuracy study, this report followed the Standards for the Reporting of Diagnostic Accuracy Studies (STARD) guidelines and checklist [18].

### 2.2. Participants

A sample of volunteers with chronic neck pain was recruited by using local announcements in two different universities located in Madrid (Spain) between May 2022 and September 2022. The eligibility criteria to allow participants to be included in the study were to be aged between 18 and 65 years old, and report mechanical neck pain for at least 3 months of duration (since this is the duration cut-off to be considered chronic neck pain) [19], with a minimum neck disability score in the Neck Disability Index of 8%. Signing the written informed consent was mandatory to participate in the study. 

Volunteers were excluded if they were in asymptomatic stages, under pharmacological treatments affecting muscle tone or pain perception, had prior history of whiplash associated disorders, neck surgeries, presented severe degenerative findings, or had any neurologic (e.g., multiple sclerosis, radiculopathy, myelopathy…), widespread pain (e.g., fibromyalgia) or medical (e.g., tumor, fractures…) condition.

### 2.3. Sample Size Calculation

Two widely used rule-of-thumb methods for estimating the minimum sample size needed were followed, as a previous study considered valid rules with enough power for detecting associations and factor analyses [20]. 

According to the recommendations provided by Green [21] for sample size estimation in regression analyses, a minimum of 50 + 8*m* (where *m* is the number of independent variables) is used. Considering that including more than 5 predictors may induce bias related with accuracy overestimation, a maximum of 5 out of the 9 predictors proposed for each model was set. Therefore, the minimum sample size required for this study was 90 participants. 

On the other hand, Harris recommended that a minimum of 10 data points per predictor variable is appropriate, resulting in at least 50 participants per model [22]. In order to avoid Type I and Type II errors, we used the model requiring the greater number of participants. 

### 2.4. Data Collection

#### 2.4.1. Sociodemographic Data

A standardized document containing age, gender, height and weight was filled out by all participants included in the study. Then, body mass index was calculated (following the formula Body Mass Index = Weight/Height^2^) [23] and included in the analyses. 

#### 2.4.2. Clinical Data

Pain intensity was measured by using the Visual Analogue Scale. Patients were asked to rate their mean pain intensity score within the last 7 days in a line of 100 mm anchored by 0 as no pain at one extreme and 100 as worst pain imaginable at the other extreme [24].

Neck disability was assessed by using the Spanish version of the Neck Disability Index since this Patient-Reported Outcome Measure (PROM) demonstrated excellent test–retest reliability (intraclass correlation coefficient = 0.98), internal consistency (Cronbach alpha = 0.89) and significant correlations with pain visual analogue scale and the Northwick Park Neck Pain Questionnaire (Pearson’s correlation coefficient = 0.65 and 0.89, respectively) [25].

This questionnaire consists of 10 items assessing disability derived from pain intensity, headache, concentration and their capacity to perform different physical activities in a 6-point Likert scale (ranging from 0 to 5). Therefore, final scores range from 0 (better health status) to 100 (greatest disability) [26].

#### 2.4.3. Cervical Multifidus Ultrasound Imaging

Two experienced examiners with +10 years of experience in musculoskeletal ultrasound imaging were involved in imaging acquisition and measurement. Each examiner acquired two images per participant (recording the left and right sides), randomizing the participants and the sides’ order, and coding each image with an alphanumeric identification. The same ultrasound equipment was used by both examiners: an Alpinion eCube i8 (Alpinion Medical systems Co, Ltd., Gyeonggi-do, Korea) with a 3–12 MHz linear transducer.

The imaging acquisition was conducted following the directives provided by Valera-Calero et al. [27] in terms of patient positioning (placed in a prone position with their arms in 90° of abduction and the elbows flexed to 90°; the head and neck stabilized using the plinth’s facial hole in a neutral position with a passive cranio-cervical movement); equipment settings (12.0 MHz of frequency; 55 dB of gain; dynamic range set to 85; brightness to 17 and depth to 4 cm); transducer placement (after manual identification of C2 spinous process and placing the transducer in this location, the transducer was caudally glided until finding the spinous process of C4, laterally glided until fixing the spinous process in the lateral extreme of the image and rotated until finding the most superficial surfaces of both the C4 spinous process and C4–C5 zygapophyseal joint, applying the minimum pressure); and cervical level (C4–C5 since this level showed the greatest agreement with MRI in comparison with other cervical levels [28]), as this procedure was tested previously in healthy [29] and clinical populations [9,14].

**Figure 1:**
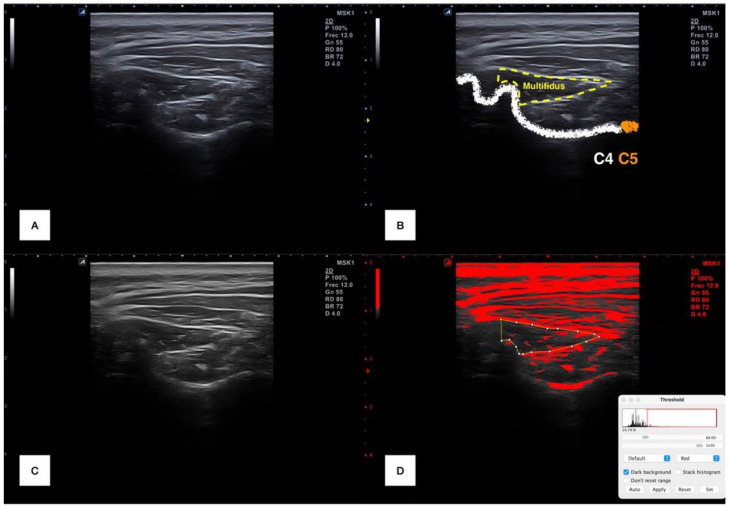
Ultrasound imaging processing. Raw image at C4–C5 level (**A**); raw image with cervical multifidus muscle delimitation (**B**); 36-bits image transformation (**C**) and selection of range of pixels for isolating the intramuscular infiltration (marked in red) (**D**).

Each examiner transferred in a DICOM format all the files to the ImageJ offline software v.1.42 (National Institutes of Health, Bethesda, MD, USA) and converted from RGB to 32-bit format (256 gray-scale format) for measuring muscle morphology and histology. First, the cervical multifidus was countered within the spinous process of C4 in the medial limit, the internal fascia between the cervical multifidus and short rotators and semispinalis in the bottom and superomedial limit, respectively. Then, a range of brightness was chosen to identify the upper cutoff echo intensity (since intramuscular infiltration is brighter than muscular tissues) for isolating the fatty infiltration, using as a reference the subcutaneous tissue that was set for each image [9,27,29,30]. Finally, muscle cross-sectional area, perimeter, mean echo intensity and fatty infiltration percentage were automatically calculated and collected. This process is illustrated in Figure 1.

### 2.5. Statistical Analyses

All statistical analyses were conducted using the SPSS v.25 for Mac OS (Armonk, NY, USA). Data normal distribution was first verified using the Shapiro–Wilk test [31]. A descriptive analysis for summarizing all demographic and muscular characteristics of the sample was conducted. Categorical data were reported as frequency and percentage (e.g., number and percentage of females and males for gender), and continuous variables as mean and standard deviation (if normally distributed) or median and interquartile range (if non-normally distributed). 

Sociodemographic characteristics were reported by gender and cervical multifidus characteristics were reported as the mean average scores of both examiners by gender (males and females) and side (right and left sides). Gender and side differences were analyzed using the Student’s T test for independent samples, considering a *p* value < 0.05 as statistically significant. Differences between gender and sides were reported as mean and 95% confidence interval. 

An inter-rater reliability was also conducted. Scores for each US variable were reported for each examiner. A mean average for each variable was also calculated and described. A 2-way mixed model, consistency type, intraclass correlation coefficient (ICC_3,2_) was calculated for reporting the agreement between both examiners. In addition, the absolute error between examiners (absolute value of the difference between both examiners), the standard error of measurement (SEM, calculated as Standard Deviation of the mean average between examiners*√1−ICC) and minimal detectable changes (MDC, calculated as 1.96×SEM×√2) [32].

Regarding the association between the absolute error for each US measurement with the demographic, clinical, muscular and histological variables, a Pearson’s correlation matrix was calculated. Correlation coefficients (r) were used to analyze the associations between variables and to identify multicollinearity and shared variance (defined as r > 0.80), to avoid the risk of bias and overestimation of the calculated model [33].

Finally, a multivariate linear stepwise regression model was calculated for each error. Variables showing the greatest correlation strength with no shared variance and statistically significant (*p* < 0.05) were included step by step. The significance criterion of the critical F value was also set at *p* < 0.05. Changes in adjusted variance (adj R^2^) were reported for each step to determine the individual variance contribution of each variable [20,21,22]. 

## 3. Results

From a total of 128 volunteers who contacted the research team to consider their participation, two were excluded since both were under pharmacological treatment with muscle relaxants at that moment. A final sample of one hundred twenty-six subjects (58 males and 68 females) was finally analyzed. Table 1 summarized the participants’ sociodemographic, clinical and cervical multifidus characteristics, and were compared by gender (sociodemographic characteristics) and by side (cervical multifidus characteristics). 

Although males were significantly (*p* < 0.001) taller and heavier than females, no significant differences were found for the Body Mass Index (*p* > 0.05). Clinical characteristics were comparable between males and females (*p* > 0.05). Regarding the cervical multifidus characteristics, no side-to-side differences were found for any of the variables assessed (*p* > 0.05), but males showed a larger cross-sectional area and perimeter and lower mean echo intensity (all, *p* < 0.001).

Interexaminer agreement is shown in Table 2. Cross-sectional area demonstrated an excellent interexaminer reliability (ICC = 0.953;0.975); perimeter (ICC = 0.855;0.928); and mean echo intensity (ICC = 0.832;0.917); a good-to-excellent reliability and the estimation of intramuscular fatty infiltration was moderate-to-good (ICC = 0.655;0.830).

**Figure 2:**
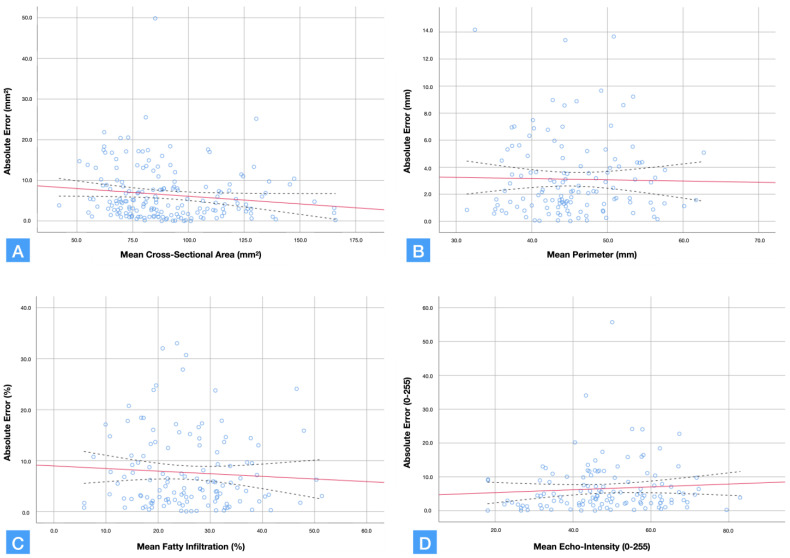
Bland–Altman plots assessing the relationship between mean scores obtained (X-axis) and the corresponding absolute error (Y-axis). Individual cases are represented with blue circles, the adjusted tendency line in red and 95% CI limits with black discontinuous lines for mean cross-sectional area (**A**), perimeter (**B**), fatty infiltration (**C**) echo-intensity (**D**) characteristics of cervical multifidus.

Additionally, the Bland–Altman plots illustrated in Figure 2 show the relationship between the mean score and the absolute error found for cross-sectional area (Figure 2A), perimeter (Figure 2B), fatty infiltration (Figure 2C) and mean echo intensity (Figure 2D).

The associations between measurements’ absolute errors with demographic, clinical, muscular and histological characteristics of the patients are reported in Table 3. Surprisingly, none of the clinical variables were shown to be associated with either the US values or the errors (all, *p* > 0.05).

In contrast, demographic characteristics showed multiple significant correlations with cervical multifidus muscle cross-sectional area, perimeter, brightness and fatty infiltration. 

Finally, the regression models explaining the contributing factors, the cross-sectional area, mean echo intensity and fatty infiltration estimation disagreement between both examiners are reported in Table 4. Although models explaining the cross-sectional area, mean echo intensity and fatty infiltration were possible to be calculated, the model for perimeter error was not possible due to the lack of significant associations. Age played the most relevant role for all the models calculated, explaining 9.6%, 10.8% and 14.0% of the fatty infiltration estimation, cross-sectional area and mean echo-intensity error variance, respectively.

## 4. Discussion

To our knowledge, this is the first study approaching the US reliability controversy regarding the assessments of deep neck extensors in clinical populations raised in the background. We aimed to assess muscular, clinical and demographic factors associated with greater disagreement; and regression analyses for each measurement were conducted. 

Previous reliability studies assessed whether US is a reliable method for assessing muscle morphology (i.e., thickness, perimeter, cross-sectional area, volume and shape descriptors, including roundness, circularity and aspect ratio) [34] and histology (i.e., mean echo intensity and intramuscular infiltration) in healthy subjects [27,29,35], and a wide range of clinical conditions including cervical radiculopathy [36], idiopathic chronic neck pain [37] and whiplash-associated disorders [9,38]. 

For instance, Kristjansson in 2004 [38] compared reliability estimates for assessing the cervical multifidus muscle in asymptomatic subjects and a small sample of female patients with whiplash-associated disorders, finding better estimates in the asymptomatic group in comparison with the cases one. The rationale provided by this author for explaining the difference was based on the loss of clarity in visualizing the fascial layer between the semispinalis cervicis and the deep neck extensors. However, this difficulty might not be just a consequence of chronic neck pain adaptations and, as demonstrated in this study, demographic characteristics may have an influence on this lack of clarity. In fact, the sample assessed by Kristjansson had a similar height, but the symptomatic group was heavier (ranging from 60 to 86 kg) in comparison with the asymptomatic group (54–78 kg). 

More recent studies, overcoming multiple limitations found in these initial studies (i.e., larger sample sizes, precise control of the examiners’ experience for the experiments and more detailed statistical processing), found acceptable reliability estimates [9,36], despite their samples being older in comparison with the sample analyzed by Kristjansson [38]. One possible reason, which was already proposed by Valera-Calero [9], is the technological development in terms of signal processing, image quality and resolution, and the quality of the transducers. However, these technological advances might not be the only reason (even if the examiners’ experience and the statistical processing are considered); since studies using updated devices still result in better reliability for asymptomatic subjects in comparison with clinical populations [9,36]. In fact, the agreement for measuring the cross-sectional area between two experienced examiners with comparable years of experience was ICC = 0.865 in patients with whiplash-associated disorders [9] and ICC = 0.965 in asymptomatic subjects [27].

Our reliability results were excellent for measuring the cross-sectional area, good-to-excellent for measuring the muscle perimeter and muscle echo intensity, and moderate-to-good for estimating the infiltration percentage. The inter-rater agreement for measuring cross-sectional area, perimeter and mean echo intensity was comparable to the results reported for asymptomatic populations [27,29]. However, the agreement for estimating the fatty infiltration percentage was worse (ICC = 0.902) [29] and more comparable to reliability estimates in whiplash-associated disorders (ICC = 0.728) [9].

Finally, several studies assessed with ultrasound age-related changes in skeletal muscles and adipose tissues, corroborating the role of age in muscle fascicle length, volume, intramuscular fat, echo intensity and thickness [39,40,41,42]. However, evidence assessing the age impact on the US measurement’s reliability is limited. We found age to be the most relevant individual contributor explaining the interexaminer disagreement for cervical multifidus US measurements, showing greater errors for calculating cervical multifidus cross-sectional area, echo intensity and fatty infiltration. Further studies may assess which age-related changes in skeletal muscles influence (to a greater extent) on the errors’ variance.

Since the asymptomatic subjects analyzed by Valera-Calero et al. [29] had larger cross-sectional areas (120 to 130 mm^2^ and 90.5 mm^2^), were younger (28.5 and 37 years old, respectively) and our results showed whether age and mean cross-sectional area are significantly associated with the fatty infiltration percentage error, this could explain the reliability results for the intramuscular infiltrates estimation. 

### Limitations

Several limitations in this study should be acknowledged. First, although we met the proposed diversity range of age, weight, height and body mass index, future studies should include samples with wider ranges of clinical severity in terms of disability, years of pain and pain intensity. This could be the reason for finding no associations between clinical characteristics and measurement errors. Secondly, just two examiners with similar experiences were involved in the study. Probably, demographic, clinical and muscular factors may influence, in a different magnitude, in examiners with less experience. Furthermore, just one piece of US equipment was used. Future studies should investigate whether different equipment or settings have an influence on measurement errors. Finally, other cervical levels should be tested.

## 5. Conclusions

This study found that US assessment of cervical multifidus at the C4–C5 level showed excellent reliability for measuring the cross-sectional area, good-to-excellent reliability for measuring the perimeter and mean echo intensity and moderate-to-good reliability for estimating the percentage of fatty infiltration in patients with mechanical chronic neck pain. Although previous evidence highlighted the role of the examiner’s experience, patient positioning, transducer placement and equipment settings, this study demonstrated whether demographic and cervical multifidus muscle characteristics influence the measurement errors in this clinical population. Future studies should consider the age of the participants, as it was the main contributor to US measurements’ error variance.

## Figures and Tables

**Table 1:** Sociodemographic, clinical and ultrasound characteristics of the sample.

Variables	Gender			Side		
	Males (n = 58)	Females (n = 68)	Difference	Left (n = 126)	Right (n = 126)	Difference
Sociodemographic Characteristics						
Age (years)	36.4 ± 11.3	37.9 ± 15.7	1.5 (−5.8; 7.2)	-	-	-
Height (m)	1.80 ± 0.06	1.64 ± 0.06	0.16 (0.14;0.18) *	-	-	-
Weight (kg)	78.1 ± 9.3	63.5 ± 11.8	14.5 (11.2;17.8) *	-	-	-
Body Mass Index (kg/m^2^)	24.1 ± 3.1	23.6 ± 4.4	0.5 (−0.8;1.7)	-	-	-
Clinical Characteristics						
Pain intensity (0–10)	4.5 ± 1.1	5.1 ± 1.2	0.5 (−0.1;1.0)	-	-	-
Pain duration (months)	4.6 ± 1.2	5.6 ± 3.4	1.0 (0.2;2.2)	-	-	-
NDI (0–100)	29.1 ± 10.2	30.0 ± 12.6	0.8 (7.9;9.2)	-	-	-
Cervical Multifidus Characteristics						
Area (mm^2^)	105.7 ± 20.8	77.5 ± 19.6	28.2 (25.1;36.6) *	91.9 ± 26.8	89.1 ± 21.0	2.8 (−2.7;7.5)
Perimeter (mm)	50.6 ± 4.7	41.0 ± 5.5	9.6 (7.7;11.7) *	45.6 ± 6.8	45.2 ± 6.3	0.4 (−1.6;2.4)
Echo Intensity (0–255)	42.3 ± 12.5	53.4 ± 13.6	11.1 (7.9;16.3) *	48.3 ± 13.1	48.3 ± 15.9	0.8 (−3.6;5.2)
Infiltration Percentage (%)	24.9 ± 6.4	26.4 ± 10.2	1.5 (−1.9;5.2)	25.9 ± 9.7	25.5 ± 9.6	0.2 (−2.6;3.3)

* Statistically significant differences (*p* < 0.001).

**Table 2:** Interexaminer reliability analysis for cervical multifidus ultrasound measurements.

Variables	Mean	Examiners		Absolute Error	ICC_3,2_ (95% CI)	SEM	MDC
		A	B				
Cross-sectional area (mm^2^)	90.5± 24.2	89.7 ± 24.4	91.2 ± 25.0	6.5 ± 6.4	0.965 (0.953; 0.975)	4.5	12.5
Perimeter (mm)	45.4 ± 6.4	44.9 ± 6.5	45.9 ± 6.9	3.1 ± 2.9	0.898 (0.855; 0.928)	2.0	5.6
Mean Echo Intensity (0–255)	48.3 ± 13.6	49.1 ± 14.1	47.5 ± 14.7	6.3 ± 6.1	0.882 (0.832; 0.917)	4.7	12.9
Infiltration Percentage (%)	25.7 ± 9.3	28.5 ± 9.9	22.9 ± 10.8	7.6 ± 7.4	0.758 (0.655; 0.830)	4.6	12.7

Abbreviatures: ICC: Intraclass correlation coefficient; MDC: Minimal Detectable Changes; SEM: Standard Error of Measurement.

**Table 3:** Pearson-Product Correlation Matrix.

	1	2	3	4	5	6	7	8	9	10	11	12	13	14
1. Age														
2. Weight	n.s.													
3. Height	−0.492 **	0.486 **												
4. BMI	0.352 **	0.800 **	−0.175 *											
5. NDI	n.s.	n.s.	n.s.	n.s.										
6. Pain intensity	n.s.	n.s.	n.s.	n.s.	0.295 *									
7. Pain duration	0.417 **	n.s.	n.s.	n.s.	0.308 *	n.s.								
8. Mean Area	−0.495 **	0.354 **	0.530 **	n.s.	0.282 *	n.s.	n.s.							
9. Mean Perimeter	−0.523 **	0.219 **	0.520 **	−0.189 *	n.s.	n.s.	n.s.	0.835 **						
10. Mean EI	n.s.	−0.453 **	−0.243 **	−0.324 *	n.s.	n.s.	n.s.	−0.324 **	−0.197 *					
11. Mean Fatty infiltration	n.s.	−0.332 **	n.s.	−0.301 **	n.s.	n.s.	n.s.	n.s.	n.s.	0.681 **				
12. Area Error	0.336 **	n.s.	−0.234 **	0.173 *	n.s.	n.s.	n.s.	n.s.	n.s.	n.s.	n.s.			
13. Perimeter Error	n.s.	n.s.	n.s.	n.s.	n.s.	n.s.	n.s.	n.s.	n.s.	n.s.	n.s.	0.205 *		
14. Mean EI Error	0.382 **	n.s.	−0.287 **	n.s.	n.s.	n.s.	n.s.	−0.265 **	−0.308 **	n.s.	n.s.	0.349 **	0.248 **	
15. Fatty infiltration Error	0.322 **	n.s.	n.s.	n.s.	n.s.	n.s.	n.s.	−0.291 **	−0.420 **	n.s.	n.s.	n.s.	n.s.	n.s.

Abbreviatures: BMI: Body Mass Index; EI: Echo Intensity; NDI: Neck Disability Index; n.s.: non-significant correlations. * *p* < 0.05; ** *p* < 0.01

**Table 4:** Summary of the regression analyses to determine individual contributors to US measurement errors.

	Predictor Outcome	*B*	SE B	95% CI	*β*	*t*	*P*
Cross-sectional area error	Step 1 Age	0.138	0.029	(0.080; 0.195)	0.336	4.710	<0.001
Mean echo-intensity error	Step 1 Age	0.171	0.037	(0.097; 0.245)	0.382	4.601	<0.001
Fatty infiltration estimation error	Step 1 Age	0.146	0.039	(0.070;0.222)	0.322	3.785	<0.001
	Step 2 Age Mean cross-sectional area	0.109 -0.063	0.043 0.032	(0.025;0.193) (−0.126;0.000)	0.240 −0.184	2.559 −1.966	0.012 0.047

Cross-sectional area: Adj R^2^ = 0.108 for step 1 (F = 22.186; *p* < 0.001); Mean echo intensity: R^2^ adj. = 0.140 for step 1 (F = 21.172; *p* < 0.001); Fatty infiltration estimation: R^2^ adj. = 0.096 for step 1 (F = 14.323; *p* < 0.001) and 0.117 for step 2 (F = 9.259; *p* < 0.001).

## Data Availability

All data derived from this study are presented in the article.
